# Epidemiological description of the demographic and HIV disease characteristics of HIV patients who are in care but not on treatment in Spain

**DOI:** 10.1186/1758-2652-13-S4-P53

**Published:** 2010-11-08

**Authors:** P Domingo, P Arazo, JL Casado, MA Castaño, E Ferrer, MA Goenaga, R Granados, F Pulido, J Rodriguez, R Rubio, J Sanz, P Viciana

**Affiliations:** 1 Hospital de Sa Santa Creu i Sant Pau, c/ Sant Antoni Maria Claret, 167, Barcelona, Spain; 2 Hospital Miguel Servet, Zaragoza, Spain; 3 Hospital Ramón y Cajal, Madrid, Spain; 4 Hospital Carlos Haya, Málaga, Spain; 5 Ciudad Univ Bellvitge, Barcelona, Spain; 6 Hospital Donostia, Donosti, Spain; 7 Hospital Dr Negrín, Las Palmas, Spain; 8 Hospital 12 de Octubre, Madrid, Spain; 9 Hospital Univ Virgen Macarena, Seville, Spain; 10 Hospital Príncipe de Asturias, Madrid, Spain; 11 Hospital Univ Virgen del Rocío, Seville, Spain

## Background

It is estimated that around 15% of HIV infected patients are being followed in HIV units for disease evolution and need of treatment. Data on the demographics and HIV disease status of these patients are scarce.

## Methods

Cross sectional analysis within 12 HIV units in Spain with the aim of describing the characteristics of patients in care but not on treatment. We collected data on demographics, HIV disease status as well as the prevalence of non-HIV co-morbidities that could qualify for antiretroviral treatment initiation regardless immunological status according to current Spanish treatment guidelines.

## Results

Data from 865 patients naïve to ARV treatment were collected. Mean age 37 y, most of them were male (83%) and Caucasian (85%). Predominant HIV risk factor was MSM (56%). Median time from HIV diagnosis 2.3 y (IQR 1.0 to 5.1). Current median CD4 cell count was 602 /mm^3^ (IQR 422 to 729) and mean HIV-1 RNA levels were 4.6 log.

The prevalence of key HIV and non-HIV features according to different CD4 strata can be seen in Table 1

**Table 1:** 

	CD4<350 (n=112)	CD4 350 - 500 (n=261)	CD4>500 (n=492)
>55 years of age	1.8%	3.1%	2.6%

HIV RNA >100,000 cop/mL	20.5%	13.4%	8.1%

CD4 percentage < 14%	19.6%	3.1%	1.2%

HCV coinfection	25.0%	14.2%	14.0%

HBV coinfection requiring treatment	0%	0.4%	0%

Liver cirrhosis	4.5%	1.1%	1.6%

HIV associated nephropaty	0%	0.8%	0%

10 y CHD>20% (Framinghan score)	Not available	Not available	0.8%

†: According to Spanish guidelines ‡: Data not available for 30% of the subjects

## Conclusions

In our cohort of patients who are under care but not on treatment, a considerable amount of them could benefit from treatment initiation according to current guidelines. When monitoring to what extent treatment guidelines are being followed, non HIV comorbidities should also be taken into account. Among these patients, HCV coinfection is the most prevalent comorbidity. Some of these patients could also benefit from generalisation of cardiovascular risk evaluation in HIV units.

